# Dietary patterns suggest that dark chocolate intake may have an inhibitory effect on oral cancer: a Mendelian randomization study

**DOI:** 10.3389/fnut.2024.1342163

**Published:** 2024-06-27

**Authors:** Hongwei Wang, Zhaoyin Zhang, Sijie Wu, Yuanzhi Zhu, Tao Liang, Xiong Huang, Jinguang Yao

**Affiliations:** ^1^Affiliated Hospital of Youjiang Medical University for Nationalities, Baise, Guangxi, China; ^2^Youjiang Medical University for Nationalities, Baise, China; ^3^Department of Tumor Pathology, The Key Laboratory of Molecular Pathology (Hepatobiliary Diseases) of Guangxi, Baise, Guangxi, China; ^4^Guilin Medical University, Guilin, Guangxi, China

**Keywords:** dark chocolate intake, sweet peppers intake, dietary intake, oral cancer, Mendelian randomization

## Abstract

**Background:**

Previous studies reported that variations in dietary intake patterns substantially impact human health, specifically tumorigenesis. However, confounding factors in previous cohort studies have obscured the relationship between dietary differences and the risk of oral cancer (OC).

**Materials and methods:**

We developed an outcome dataset from genome-wide association studies (GWAS) data on three OCs within the GAME-ON project, using GWAS-META merging. We extracted 21 dietary exposures, including 10 dietary patterns, 6 vitamins, and 5 micronutrients, from the UK Biobank database, using the inverse variance weighting method as the primary statistical method. Sensitivity analysis was conducted to detect heterogeneity and pleiotropy. Serum metabolite concentrations were adjusted using multivariate Mendelian randomization.

**Results:**

Of the 10 analyzed dietary patterns, 8 showed no significant association with the risk of developing OC. Consumption of dark chocolate (inverse variance weighted [IVW]: Odds ratio (OR) = 0.786, 95% confidence interval [CI]: 0.622–0.993, *p* = 0.044) and sweet pepper exhibited an inverse relationship with OC risk (IVW: OR = 0.757, 95% CI: 0.574–0.997, *p* = 0.048). Reverse MR analysis revealed no reverse causality. Furthermore, no significant correlation was observed between the intake of 6 vitamins and 5 micronutrients and the risk of developing OC. After using multivariable MR to adjust for serum caffeine, linoleate, theophylline, and theobromine metabolism levels, consuming dark chocolate was unrelated to a decreased risk of OC. After adjusting each serum metabolite individually, the observed *p*-values deviated from the original values to varying degrees, indicating that the components of dark chocolate could have different effects. Among these components, theophylline demonstrated the most significant inhibitory effect.

**Conclusion:**

This study demonstrated a causal relationship between the intake of dark chocolate and sweet peppers and a lower risk of OC. The components of dark chocolate could have different effects.

## 1 Introduction

Oral cancer (OC) is a common malignancy of the head and neck region. Globally, it accounts for approximately 405,000 new cases annually (1). Tobacco use and alcohol consumption are well-documented etiological factors of OC (2, 3). In China, betel nut consumption is a common etiological factor for OC (4). An OC usually has no apparent symptoms in the early stages, making early and prompt diagnostic intervention and routine medical evaluations difficult. Patients with advanced stages of OC frequently present with symptoms, including ulcers, lumps, and lymph node metastases in the neck region. Consequently, their chances of survival at the time of diagnosis are significantly reduced (5). Numerous strategies for early OC screening have been developed to facilitate earlier detection and treatment. However, there is a critical need for more precise and effective screening approaches (6). Various dietary intakes are implicated in disease development (7, 8). Therefore, clarifying the relationship between various dietary intakes and OC is crucial to preventing disease and changing the lifestyle habits of high-risk populations.

The degree to which dietary intake affects OC remains unknown (9). Unlike single-nutrient studies that are often influenced by various confounding factors, studies of dietary intake patterns offer a more accurate reflection of the body’s condition in everyday life by summarizing and analyzing individual nutrients (10). This study employed the Mendelian randomization (MR) method to clarify these potential associations and comprehensively investigate the plausible link between various diets and OC.

The MR is a method to investigate possible associations between an exposure factor (dietary intake) and an outcome (OC). The association between exposure and outcome was analyzed at the genetic level using data sourced from public databases. One major advantage of MR is its ability to reduce potential confounders in clinical research and substantially reduce the possibility of reverse causation (11). This improves the precision, reliability, and validity of the findings.

Food often contains multiple components that can have individual or synergistic effects. Investigating the primary components of a food may be more effective in determining its mechanism of action. Although MR analysis has numerous benefits, it cannot identify the roles of individual components because of its inherent limitations. Conversely, multivariable MR (MVMR) is a method for investigating the direct or mediated effects of two or more exposures on outcomes (12). Consequently, by considering each internal component of a nutrient, MVMR enables a direct assessment of its impact on an outcome, making it easier to investigate the role of each component.

### 1.1 Method design

This study used a two-sample MR design to investigate the relationship between dietary intake and OC. In MR studies, single nucleotide polymorphisms (SNPs) utilized as instrumental variables (IVs) must satisfy three core criteria (11): First, SNPs must be strongly associated with the exposure factors. Second, SNPs should not be associated with confounding variables other than exposure factors. Third, SNPs must not be directly associated with the outcome, in this case, OC. Moreover, reverse MR analysis was employed to investigate the potential reverse causality effects of OC on positive outcomes. Additionally, MVMR was used to determine the direct impact of positive exposures on OC.

### 1.2 Data source

This study included 21 exposure factors, including 10 dietary intakes (oily fish, non-oily fish, alcohol, tea, coffee, cooked vegetables, salad/raw vegetables, fresh fruit, dark chocolate, and sweet pepper), 6 vitamins (vitamin B12, vitamin C, vitamin B6, vitamin D, vitamin E, and carotene), and 5 trace minerals (zinc, copper, calcium, magnesium, and potassium). Specific information about the diet in our study can be found in the publicly available information on the UK Biobank.^1^ We combined genome-wide association studies (GWAS) data on OC from three geographic regions using a GWAS-meta-analysis approach. The data obtained from the GAME-ON consortium included 2,989 cases and 6,583 controls, with >90% of participants predominantly of European ancestry (>70% CEU) (13). Furthermore, Chen et al. (14) and the GWAS catalog^2^ provide serum metabolite levels of theobromine, theophylline, caffeine, and linoleate (18:2n6). Other GWAS datasets are available through the Integrative Epidemiology Unit Open GWAS project.^3^ Table 1 shows the details of all the data.

**TABLE 1:** The GWAS data source details in our study.

Phenotype	Data source	GWAS ID	PMID	Case	Control	Sample size	Ancestry
Oral cancer	GAME-ON	NA	27749845	2989	6583	9572	>90% of participants were European
Non-oily fish intake	MRC-IEU	ukb-b-17627	NA	NA	NA	460880	European
Oily fish intake	MRC-IEU	ukb-b-2209	NA	NA	NA	460443	European
Alcohol intake frequency.	MRC-IEU	ukb-b-5779	NA	NA	NA	462346	European
Tea intake	MRC-IEU	ukb-b-6066	NA	NA	NA	447485	European
Coffee intake	MRC-IEU	ukb-b-5237	NA	NA	NA	428860	European
Salad / raw vegetable intake	MRC-IEU	ukb-b-1996	NA	NA	NA	435435	European
Fresh fruit intake	MRC-IEU	ukb-b-3881	NA	NA	NA	446462	European
Cooked vegetable intake	MRC-IEU	ukb-b-8089	NA	NA	NA	448651	European
Dark chocolate intake	MRC-IEU	ukb-b-16139	NA	NA	NA	64945	European
Sweet pepper intake	MRC-IEU	ukb-b-10932	NA	NA	NA	64949	European
Vitamin B12	MRC-IEU	ukb-b-19524	NA	NA	NA	64979	European
Vitamin C	MRC-IEU	ukb-b-19390	NA	NA	NA	64979	European
Vitamin B6	MRC-IEU	ukb-b-7864	NA	NA	NA	64979	European
Vitamin D	MRC-IEU	ukb-b-18593	NA	NA	NA	64979	European
Vitamin E	MRC-IEU	ukb-b-6888	NA	NA	NA	64979	European
Carotene	MRC-IEU	ukb-b-16202	NA	NA	NA	64979	European
Zinc	NA	ieu-a-1079	23720494	NA	NA	2603	European
Copper	NA	ieu-a-1073	23720494	NA	NA	2603	European
Calcium levels	NA	ebi-a-GCST90025990	34226706	NA	NA	400792	European
Magnesium	MRC-IEU	ukb-b-7372	NA	NA	NA	64979	European
Potassium	MRC-IEU	ukb-b-17881	NA	NA	NA	64979	European
Theobromine levels	NA	GCST90199644	36635386	NA	NA	8137	European
Theophylline levels	NA	GCST90199647	36635386	NA	NA	7822	European
Caffeine levels	NA	GCST90200436	36635386	NA	NA	8005	European
Linoleate (18:2n6) levels	NA	GCST90200354	36635386	NA	NA	8260	European

### 1.3 Selection of instrumental variables

We identified SNPs associated with exposure factors that met a significance threshold (*p* < 5 × 10^–6^). SNPs were selected to be free of linkage disequilibrium to ensure independence, with an r^2^ < 0.001 within a 10,000 KB window. Surrogate SNPs were not considered. SNPs with F-statistic values <10 were regarded as weak IVs and were excluded. If the SNP was not found in the resulting GWAS dataset, it was excluded. Subsequently, we estimated that positive stranded alleles and SNPs with palindromes were eliminated. The qualifying SNPs were utilized in the subsequent analytical phase. The conditions and protocols mentioned above were used for inverse MR and MVMR analyses.

### 1.4 Statistical analysis

In our MR analysis, we employed five approaches: inverse variance weighted (IVW), weighted median, MR-Egger, weighted mode, and simple mode. These approaches aimed to investigate the potential association between dietary intake and the risk of developing OC. The value of *p* < 0.05 was deemed to be statistically significant. The directional consistency of associations, as represented by the inverse variance weighted IVW, weighted median, and MR-Egger methods, proved to be useful. The IVW was selected as the primary reference due to its excellent accuracy in identifying causality (15). The MR-Egger method was particularly responsive to horizontal pleiotropy and heterogeneity in the outcomes. The other three outcomes, weighted median, and two more served as supplementary approaches to the MR analysis. Results with inconsistent odds ratios (ORs) or evidence of horizontal pleiotropy were excluded from the analyses performed using the three MR methods: IVW, MR-Egger, and weighted median. The ORs were used to assess the impact of dietary factors on the risk of OC.

## 2 Results

**FIGURE 1:**
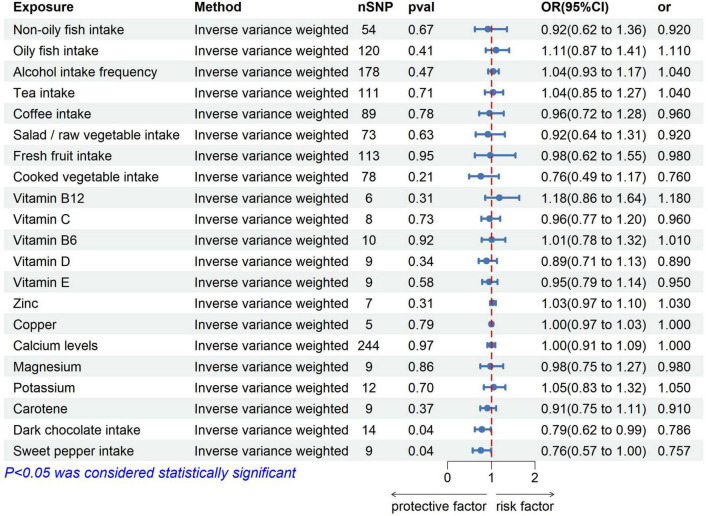
Inverse variance weighted results of MR for 21 dietary intakes and OC.

**Two-sample MR results:** We used five MR statistics to assess the association between 21 exposures and the risk of OC. Figure 1 depicts the IVW results for the 21 exposures.

Of the 10 dietary patterns analyzed, 8 dietary intakes showed no significant association with OC risk (Supplementary Table 1). However, dark chocolate intake was inversely associated with OC risk (IVW: OR = 0.786, 95% confidence interval [CI]: 0.622–0.993, *p* = 0.044). Furthermore, the MR-Egger intercept test did not indicate evidence of horizontal pleiotropy (*p* > 0.05) (Supplementary Figure 1). The consistency of these findings was further confirmed by the leave-one-out method, suggesting the stability of the results (Supplementary Figure 2).

Similarly, sweet pepper intake was found to reduce the risk of OC (IVW: OR = 0.757, 95% CI: 0.574–0.997, *p* = 0.048). The MR-Egger intercept test did not indicate the presence of horizontal pleiotropy (*p* > 0.05) (Supplementary Figure 3). Moreover, the leave-one-out analysis revealed no outliers (Supplementary Figure 4). For both significant findings, the direction of the ORs obtained through the other three MR methods was consistent. This indicated that the results were more accurate (Table 2).

**TABLE 2:** Results of MR analysis of Dark chocolate intake and Sweet pepper intake with oral cancer.

Exposure	Outcome	SNP	Method	OR	95% CI	P-value	egger_intercept	Q_df(Q_pval)
Dark chocolate intake	Oral cancer	14	MR Egger	0.759	0.487 - 1.179	0.244	0.002	13 (0.838)
			Weighted median	0.739	0.543 - 1.005	0.054		12 (0.781)
			Inverse variance weighted	0.786	0.622 - 0.993	**0.044**		
			Simple mode	0.71	0.50 - 1.01	0.08		
			Weighted mode	0.75	0.55 - 1.01	0.08		
Sweet pepper intake	Oral cancer	9	MR Egger	0.598	0.325 - 1.100	0.142	0.012	8 (0.612)
			Weighted median	0.694	0.472 - 1.018	0.062		7 (0.588)
			Inverse variance weighted	0.757	0.574 - 0.997	**0.048**		
			Simple mode	0.65	0.39 - 1.10	0.15		
			Weighted mode	0.70	0.47 - 1.04	0.12		

MR, Mendelian randomization; OR, odds ratio; 95% CI, 95% confidence interval; Heterogeneity, Heterogeneity Test; Pleiotropy, Pleiotropy Test; Q_pval, horizontal pleiotropy *P*-value for Q-test.

Regarding the other 6 vitamins (vitamin B12, vitamin C, vitamin B6, vitamin D, vitamin E, and carotene) and 5 trace elements (zinc, copper, calcium, magnesium, and potassium), the MR analysis indicated that the intake of these nutrients was not associated with an increased risk of OC (Supplementary Table 1).

**Reverse MR results:** We conducted a reverse MR analysis to determine whether the intake of dark chocolate and sweet pepper affects the risk of developing OC. The analysis revealed that dietary intake does not inversely affect OC development. Furthermore, the sensitivity analysis revealed no evidence of heterogeneity or pleiotropy. The leave-one-out analysis demonstrated consistent results, highlighting the stability of our findings (Supplementary Figures 5, 6 and Supplementary Table 2).

**MVMR results:** Given that numerous components in dark chocolate are known to have direct or indirect cancer-inhibitory bioactivity (16), we aimed to investigate their impact on oral carcinogenesis. Although trace elements in dark chocolate are known as active components (16), our two-sample MR analysis demonstrated that these factors do not influence the development of OC, leading to their exclusion from further analysis. Consequently, we employed MVMR to adjust serum metabolite levels for caffeine, linoleate, theophylline, and theobromine. This analysis indicated that dark chocolate intake was not associated with the risk of developing OC (Table 3).

**TABLE 3:** Multivariable MR results of causal links between Dark chocolate and oral cancer after adjusting for specific serum metabolites.

Exposure	Outcome	Adjustment of metabolites	Method	Beta	Se	*P*-value	OR	95% CI	Heterogeneity test	Egger (intercept)
Dark chocolate	Oral cancer	Caffeine levels	MVMR-IVW	−0.2	0.146	0.168	1.22	0.61–1.10	0.1896
		Linoleate levels							
		Theobromine levels	MVMR-Egger	−0.134	0.167	0.422	0.87	0.55–1.20	0.1826	0.397
		Theophylline levels								

MR, Mendelian randomization; OR, odds ratio; 95% CI, 95% confidence interval; Heterogeneity, Heterogeneity Test; Pleiotropy, Pleiotropy Test.

We adjusted each ingredient separately in the MVMR analysis to elucidate the role of these factors in the suppression of OC by dark chocolate. After adjusting for each serum metabolite individually, we observed a decrease in *p*-values compared to the original values, specifically when adjusting for linoleate levels. This suggested a potential reduction in the inhibitory effect of dark chocolate on OC associated with linoleate levels. Conversely, adjusting for theobromine levels or theophylline levels resulted in higher *p-*values than the original values, with theophylline levels demonstrating a more pronounced increase. These findings suggest that theophylline may be the primary inhibitory factor in dark chocolate, while theobromine has a relatively limited inhibitory effect (Table 4).

**TABLE 4:** Multivariable MR results of causal links between Dark chocolate and oral cancer after adjusting for single serum metabolites.

Exposure	Outcome	Adjustment of metabolites	Method	Beta	Se	*P-* value	OR	95% CI
Dark chocolate	Oral cancer	Caffeine levels	MVMR-IVW	−0.22	0.11	0.04	0.8	0.65–0.99
Dark chocolate	Oral cancer	Linoleate levels	MVMR-IVW	−0.24	0.11	0.02	0.78	0.64–0.97
Dark chocolate	Oral cancer	Theobromine levels	MVMR-IVW	−0.23	0.12	4.80E−02	0.79	0.63–1.00
Dark chocolate	Oral cancer	Theophylline levels	MVMR-IVW	−0.24	0.15	9.93E−02	0.79	0.59–1.05

MVMR, multivariate Mendelian randomization; OR, odds ratio; IVW, Inverse Variance Weighted; 95% CI, 95% confidence interval.

Despite our efforts to understand how sweet pepper may influence oral carcinogenesis, limitations in data availability restricted further investigation in this area.

## 3 Discussion

We found that dark chocolate and sweet pepper intake may exert a significant inhibitory effect on the development of OC. The MR analysis did not reveal any considerable association between OC risk and the remaining 8 dietary intakes (oily fish, non-oily fish, alcohol, tea, coffee, cooked vegetables, salad/raw vegetables, fresh fruit), 6 vitamins (vitamin B12, vitamin C, vitamin B6, vitamin D, vitamin E, and carotene), and 5 trace minerals (zinc, copper, calcium, magnesium, and potassium).

Considering the variety of vitamins and micronutrients in the daily diet, pooling and analyzing individual nutrients can significantly help eliminate the confounding factors often present in studies focused on specific nutrients. The findings of various studies on the association between dietary intake and OC are inconsistent (9). Concerning fish consumption, our findings are consistent with those of previous studies (17–19), which found no clear association between oily and non-oily fish intake and the development of OC. Although alcohol is considered potentially carcinogenic (20), its association with OC is unknown (21). Furthermore, the impact of tea and coffee consumption on OC is controversial (22). Our findings imply that their role may not be considerable, which is consistent with the findings of previous studies conducted by Tverdal et al. (23) and Hildebrand et al. (24). Fruits and vegetables, which are rich in vitamins, enhance antioxidant and anti-inflammatory responses, potentially inhibiting tumor growth (25). However, our findings did not demonstrate any substantial inhibitory effect of cooked vegetables, salad/raw vegetables, or fresh fruit intake on OC, inconsistent with the findings of previous studies (26, 27). Moreover, our analysis of vitamins and OC revealed no association between the intake of six common vitamins and the risk of OC, confirming our findings. Furthermore, although numerous studies (28) have highlighted the health benefits of micronutrients, our study did not find any substantial role of micronutrients in the risk of OC. We believe that the inconsistency between our study’s results and those of other experiments can be attributed to the following factors: 1. Previous observational studies may contain confounding factors, whereas our study, based on Mendelian Randomization (MR) analysis of Genome-Wide Association Studies (GWAS), minimizes the effects of confounding and reverse causation. 2. Our study is limited to European ancestries only. Variations in ancestry can influence disease susceptibility, thereby contributing to differences in results. Our findings suggest that while no direct association was found, dietary intake may still have some influence on the development of OC. These findings require further validation in future research.

Our findings indicated that dark chocolate intake may have a significant inhibitory effect on OC. Currently, studies that specifically investigate the impact of dark chocolate on OC are rare. Although some studies have proposed that various components of dark chocolate, including micronutrients, can have varying effects on overall health (16), their specific implications for OC are unknown. In this study, MVMR analysis suggested that caffeine, linoleate, theophylline, and theobromine levels may each have distinct roles in inhibiting oral carcinogenesis associated with consuming dark chocolate, particularly when controlling for several common components. Cocoa, the main ingredient in dark chocolate, contains a high percentage of methylxanthine compounds, mainly theobromine and caffeine (29). These substances are known for their potent antioxidant effects, aiding in the scavenging of free radicals, reducing DNA damage and oxidative stress, and thus potentially preventing cancer at its onset (30). Contrary to our initial expectations, our MVMR analysis revealed that caffeine in dark chocolate may not inhibit OC as anticipated. Furthermore, while theobromine has demonstrated inhibitory effects on OC, these effects are limited. Notably, theophylline in dark chocolate demonstrated a stronger inhibitory effect despite the lower percentage of theophylline in cocoa (31). Theophylline, a naturally occurring methylxanthine, is known to impact adenosine activation and downstream signaling pathways of cAMP by inhibiting cAMP phosphodiesterase activity (32). The activation of the cAMP signaling pathway plays a crucial role in the proliferation and growth of cancer cells. Notably, theophylline also inhibits the activation of NF-κB and the release of inflammatory factors such as IL-6 (33), which are implicated in creating a tumor-favorable inflammatory microenvironment that promotes cancer development and progression. Additionally, in breast cancer, theophylline has been shown to induce cell cycle arrest in the G2/M phase through the phosphorylation of cell cycle proteins B1 and Cdc2 (34). Therefore, it is evident that theophylline plays a distinct role in cancer inhibition, which aligns with the findings of our study. Dark chocolate contains a high percentage of linoleate (18:2n6) (35). These linoleic acids decrease cholesterol levels of low-density lipoproteins and fasting blood glucose concentrations by improving insulin sensitivity (36, 37). Metabolic disorders, including diabetes mellitus and obesity, are associated with an increased risk of oral carcinogenesis (38, 39). Contrary to the anticipated findings, our findings demonstrated that the inhibitory effect of dark chocolate on OC was reduced after adjusting for linoleate levels. This suggests that linoleate levels might not fulfill their anticipated role but rather may slightly reduce the inhibitory effect of dark chocolate. Further studies are required to clarify its exact role in the impact of dark chocolate.

The above findings suggest that the biological activity of dark chocolate is not attributable to a single ingredient. Furthermore, dark chocolate is specifically abundant in total polyphenols and flavonoids (40), which are known to have antioxidant properties. These may be crucial in inhibiting the development of OC (41, 42). However, the data restrictions prevented us from fully investigating and analyzing this aspect. Additionally, dark chocolate intake has been associated with improved mood (43), suggesting an additional psychological benefit. Our findings indicate that the consumption of sweet pepper may substantially inhibit the development of OC. Sweet pepper are rich in an array of phytochemicals including polyphenols, flavonoids, carotenoids, capsaicinoids, and dihydrocapsaicin. Han et al. (44) observed that capsaicinoids inhibit the activation of the NF-κB pathway and activator protein-1 (AP-1), suggesting a potential role in cancer and inflammation prevention. Further research indicates that capsaicin activates the AMPK/mTOR signaling pathway to induce autophagy in renal cancer cells, thereby reducing tumor proliferation, invasion, and epithelial-mesenchymal transition (EMT) (45). Additionally, capsaicin facilitates the phosphorylation of extracellular signal-regulated kinase (ERK) and c-Jun, while inhibiting the Hedgehog/GLI pathway, exhibiting antiproliferative and anticancer properties (46). In cases of oral cancer, capsaicin has been shown to suppress tumor cell proliferation and induce apoptosis. (47). Interestingly, capsiate, another compound in sweet pepper, is effective in inhibiting tumor activity (48). Despite our efforts to further understand the underlying mechanisms, the limitations of our data prevented a more in-depth investigation.

This is a novel study on the association between various dietary patterns and OC using multiple MR analysis methods. To our knowledge, this is the first study to uncover this relationship through MR. The lack of studies that examine the relationship between dietary intake and OC highlights the importance of our findings. These findings lay the foundation for future larger-scale clinical studies and basic research to investigate this association more thoroughly. Moreover, our research bridges the gap in understanding the association between dark chocolate and OC risk. It investigates the potential mechanisms by which dark chocolate components can suppress OC, laying the foundation for future fundamental research. Our results provide insights into the role of different dietary intakes in the development of OC, offering guidance for individuals at elevated risk of OC to adjust their diets in a clinically meaningful way.

There are limitations to our study. First, to capture a wide range of associations between dietary variety and OC, we employed a significance threshold of *p* < 5 × 10^–6^ rather than the more conventional *p* < 5 × 10^–8^. Although our datasets were solely of European origin, it is crucial to conduct further studies that include diverse regions to investigate these associations globally. Second, the nature of the MR analysis prevented us from determining a dose-response relationship between various intakes and OC at this stage. Third, we did not categorize outcomes by clinical, pathological, site, or other clinical features to investigate the relationship between diet and each subtype. While we have identified possible mechanisms of action for dark chocolate, limited data prevented definitive conclusions on the role of all its ingredients.

## 4 Conclusion

This study demonstrated a causal association between the intake of dark chocolate and sweet peppers and a decreased risk of OC. The components of dark chocolate could have different effects.

## Data availability statement

All GWAS data are available by accessing the open GWAS database (https://gwas.mrcieu.ac.uk/) or GWAS Catalog (https://www.ebi.ac.uk/gwas/), further inquiries can be directed to the corresponding author.

## Ethics statement

All data for the study were obtained from publicly available databases that had been approved by the Ethics Committee, so no additional information was required. The studies were conducted in accordance with the local legislation and institutional requirements.

## Author contributions

HW: Conceptualization, Methodology, Formal analysis, Visualization, Writing – original draft. ZZ: Conceptualization, Software, Visualization, Writing – original draft. SW: Conceptualization, Data curation, Validation, Writing – original draft. YZ: Writing – original draft. TL: Formal analysis, Validation, Writing – original draft. XH: Resources, Data curation, Writing – original draft. JY: Writing review and editing, Supervision, Project administration.

## Footnotes

1
https://biobank.ctsu.ox.ac.uk/crystal/label.cgi?id=100052

2
https://www.ebi.ac.uk/gwas/

3
https://gwas.mrcieu.ac.uk/
