# Supplementary figures and images for: Dietary patterns suggest that dark chocolate intake may have an inhibitory effect on oral cancer: a Mendelian randomization study
Source: Front Nutr. 2024 Jun 27;11:1342163. doi: 10.3389/fnut.2024.1342163 (PMC11255456; doi:10.3389/fnut.2024.1342163)

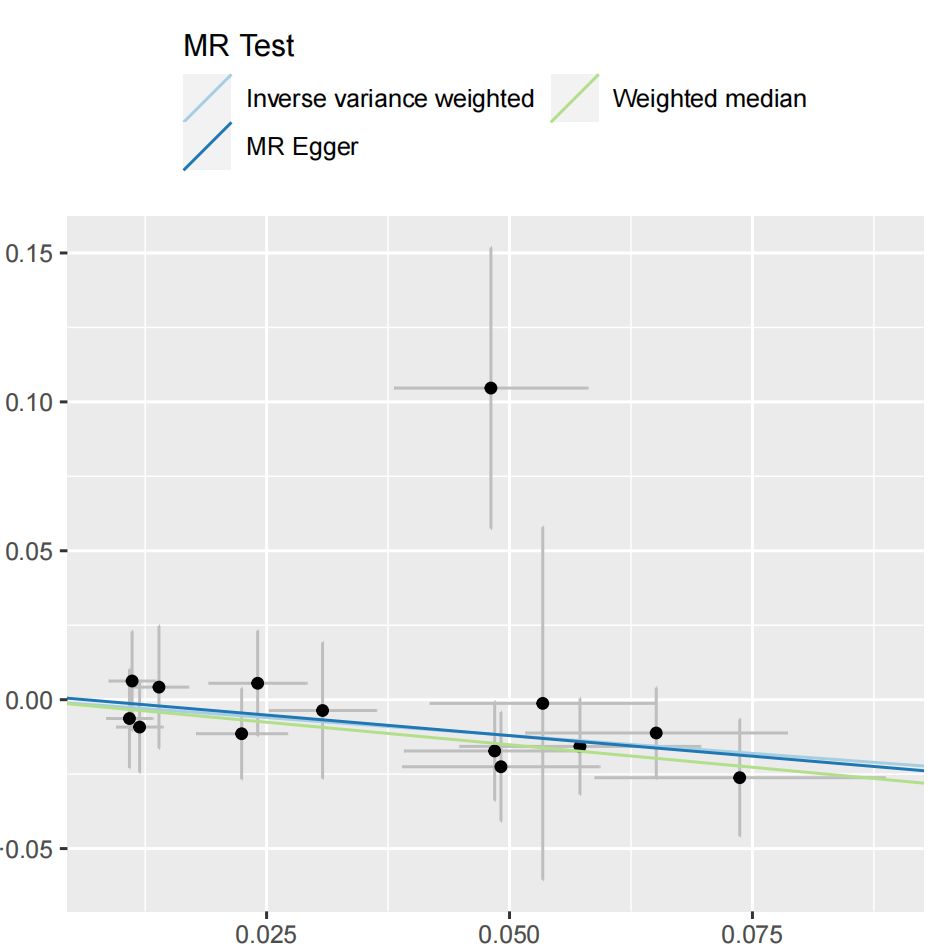

Supplement: Supplementary file 3 [file Image_1.jpeg]

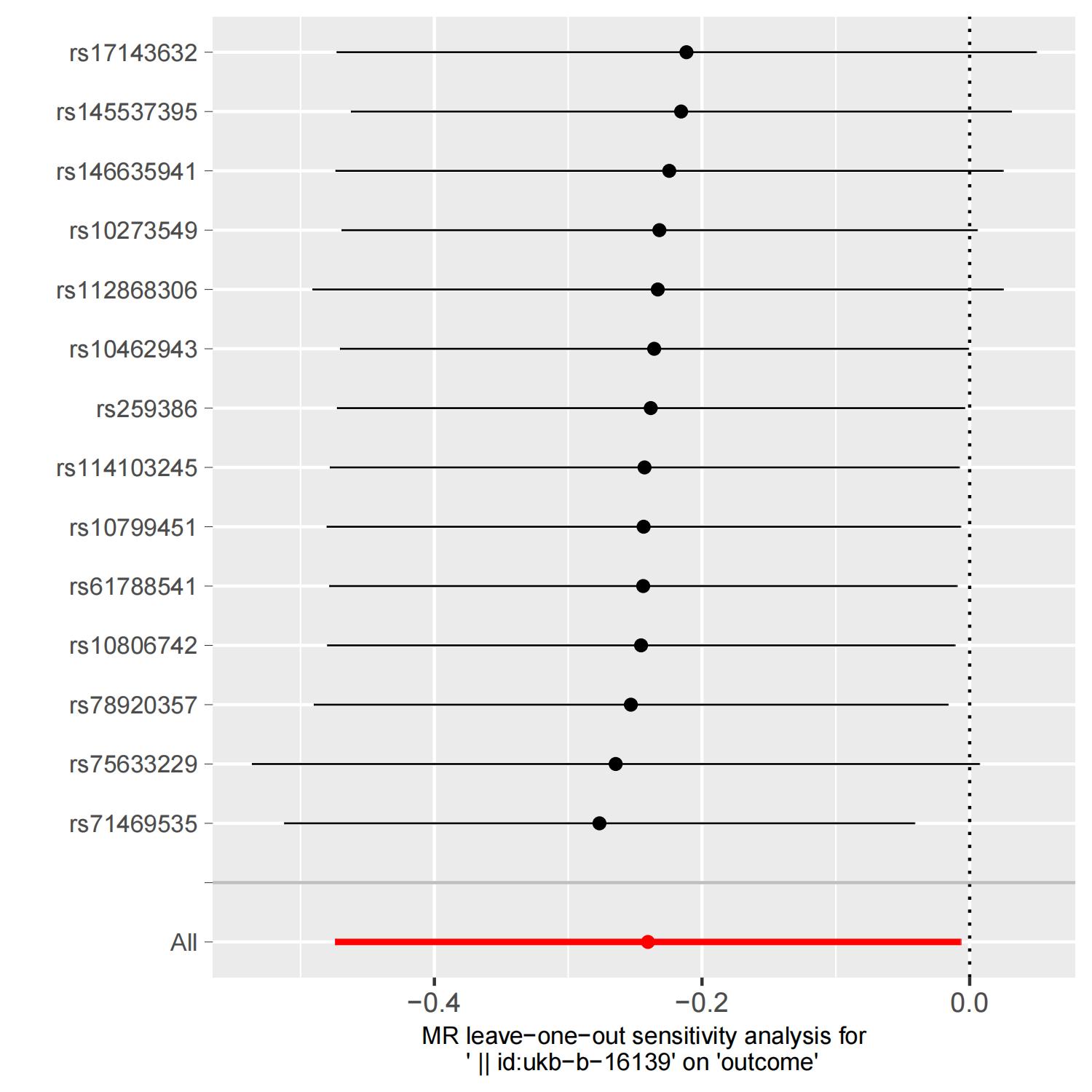

Supplement: Supplementary file 4 [file Image_2.jpeg]

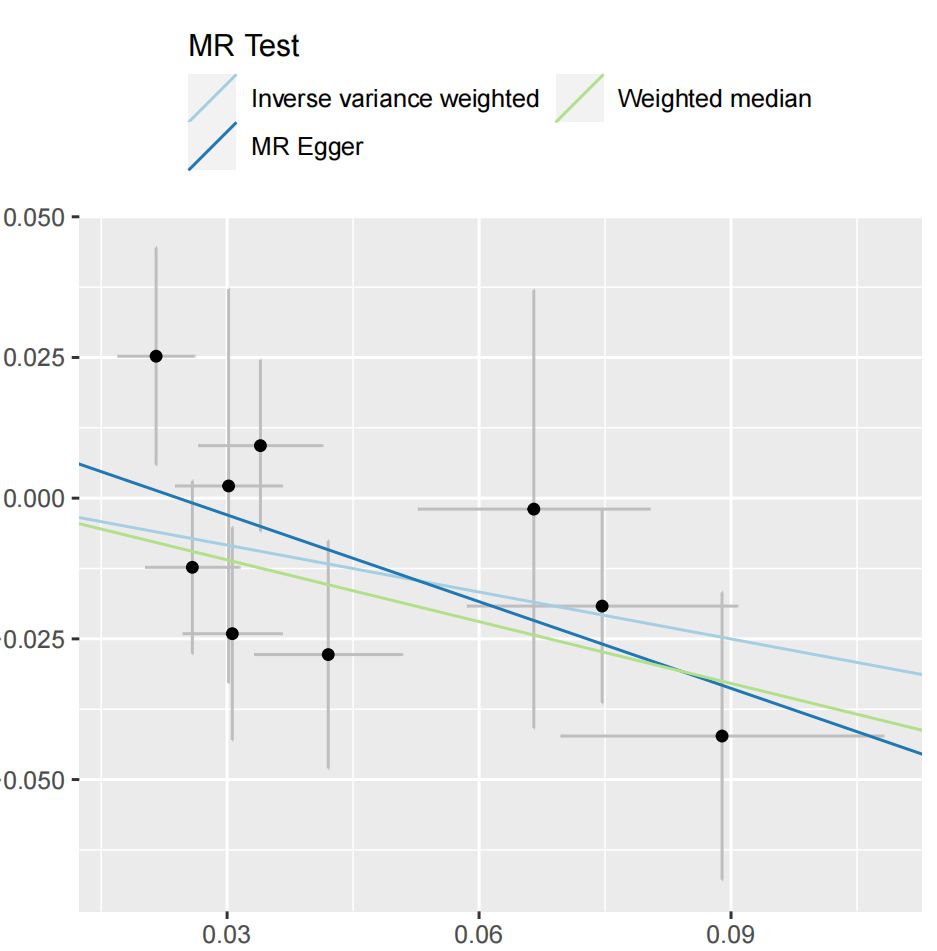

Supplement: Supplementary file 5 [file Image_3.jpeg]

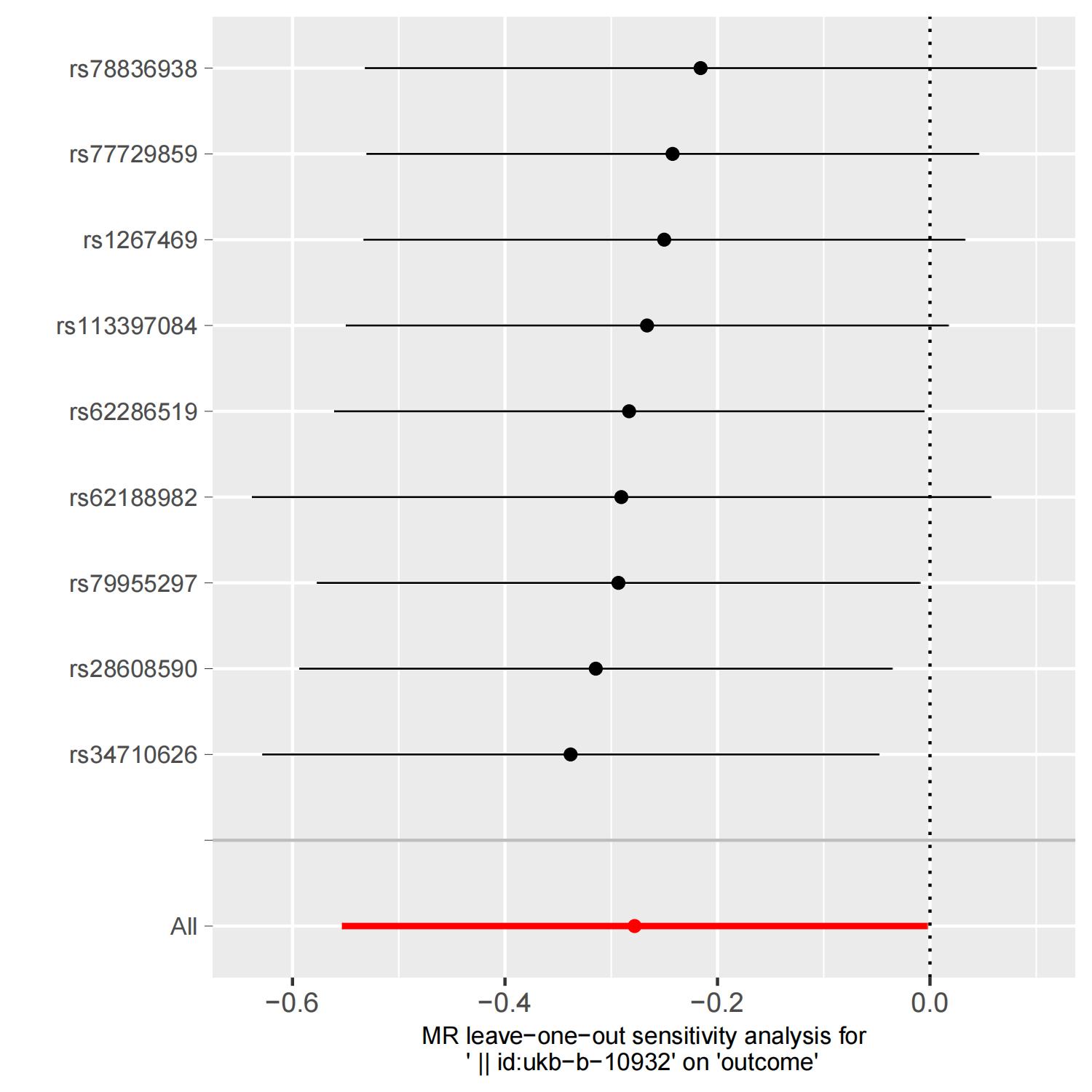

Supplement: Supplementary file 6 [file Image_4.jpeg]

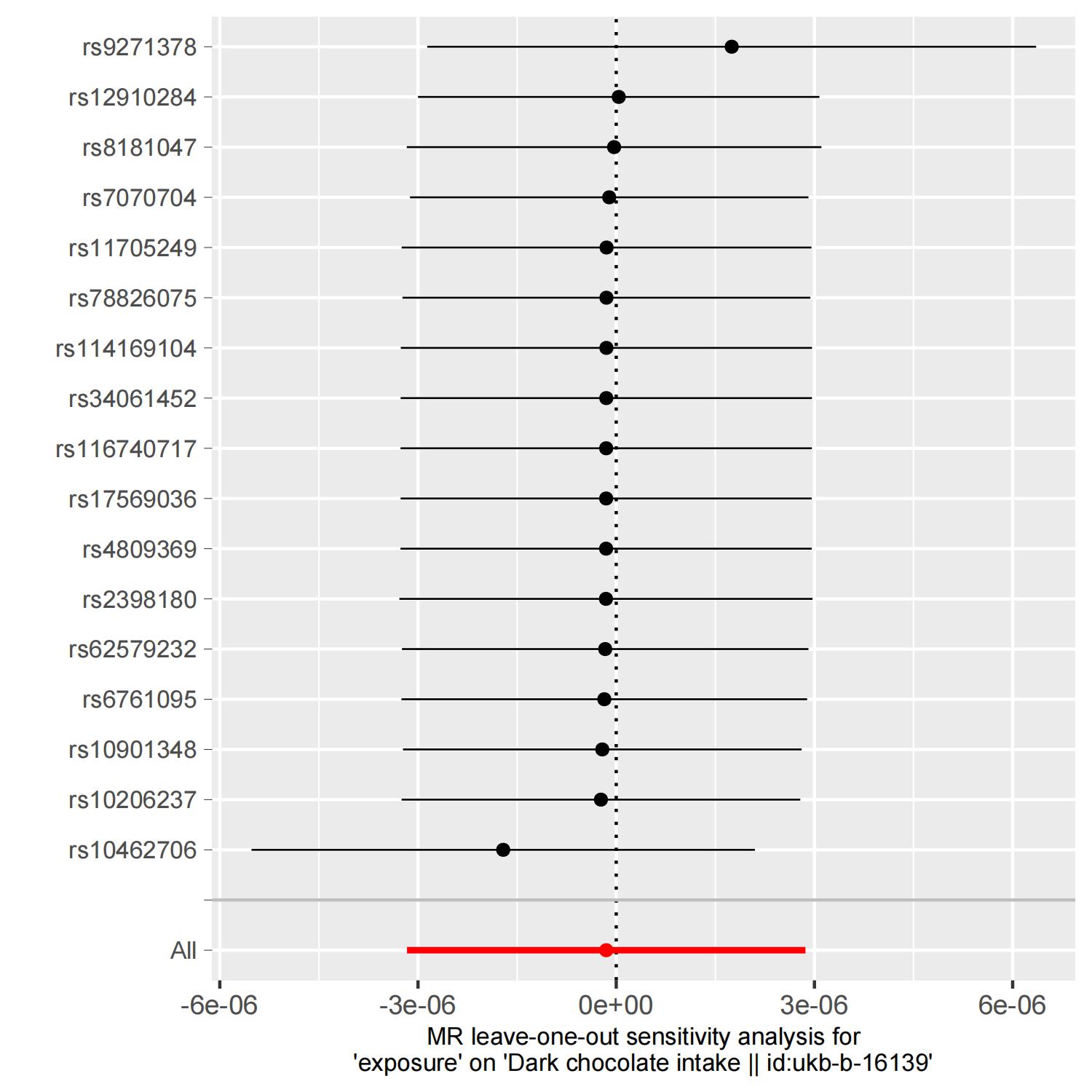

Supplement: Supplementary file 7 [file Image_5.jpeg]

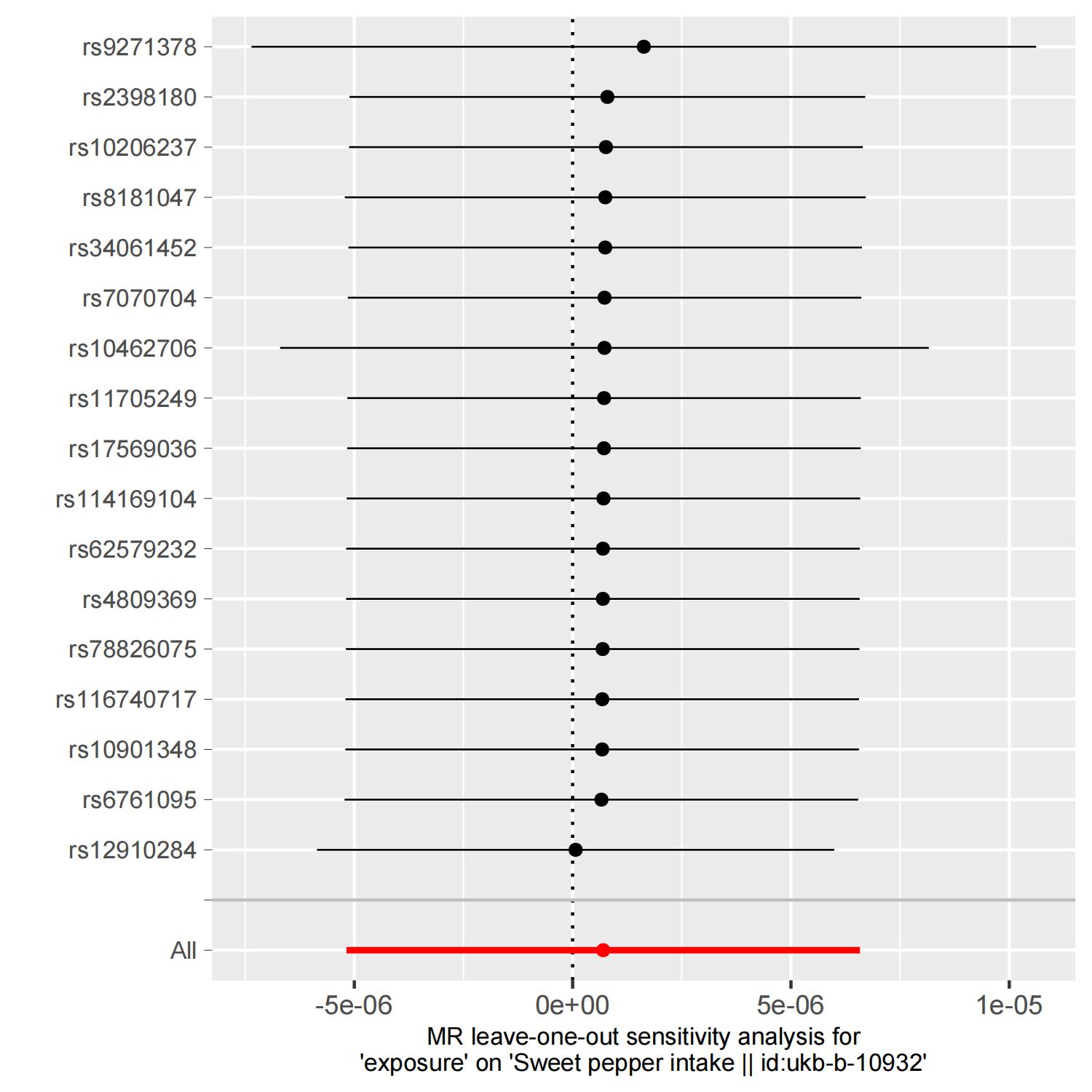

Supplement: Supplementary file 8 [file Image_6.jpeg]
